# EPO‐receptor is present in mouse C2C12 and human primary skeletal muscle cells but EPO does not influence myogenesis

**DOI:** 10.1002/phy2.256

**Published:** 2014-03-26

**Authors:** Séverine Lamon, Evelyn Zacharewicz, Andrew N. Stephens, Aaron P. Russell

**Affiliations:** 1 Centre for Physical Activity and Nutrition, School of Exercise and Nutrition Sciences, Deakin University, Burwood, Victoria, Australia; 2 Prince Henrys Institute for Medical Research, Clayton, Victoria, Australia

**Keywords:** Cytokine, erythropoietin, erythropoietin‐receptor, myogenesis, skeletal muscle

## Abstract

The role and regulation of the pleiotropic cytokine erythropoietin (EPO) in skeletal muscle are controversial. EPO exerts its effects by binding its specific receptor (EPO‐R), which activates intracellular signaling and gene transcription in response to internal and external stress signals. EPO is suggested to play a direct role in myogenesis via the EPO‐R, but several studies have questioned the effect of EPO treatment in muscle in vitro and in vivo. The lack of certainty surrounding the use of nonspecific EPO‐R antibodies contributes to the ambiguity of the field. Our study demonstrates that the EPO‐R gene and protein are expressed at each stage of mouse C2C12 and human skeletal muscle cell proliferation and differentiation and validates a specific antibody for the detection of the EPO‐R protein. However, in our experimental conditions, EPO treatment had no effect on mouse C2C12 and human muscle cell proliferation, differentiation, protein synthesis or EPO‐R expression. While an increase in Akt and MAPK phosphorylation was observed, we demonstrate that this effect resulted from the stress caused by changing medium and not from EPO treatment. We therefore suggest that skeletal muscle EPO‐R might be present in a nonfunctional form, or too lowly expressed to play a role in muscle cell function.

## Abstract (precis)

The EPO‐R is expressed at the gene and protein level in mouse and human myoblasts and myotubes. However, EPO treatment does not seem to activate the EPO‐R and its downstream signaling pathways in skeletal muscle cells, questioning its functionality.

## Introduction

Erythropoietin (EPO) is a cytokine hormone primarily dedicated to erythroid progenitor cell proliferation and development. EPO exerts its effect by binding its specific trans‐membrane receptor, the EPO‐receptor (EPO‐R; D'Andrea and Zon 1990; Youssoufian et al. 1993). In erythroid progenitor cells, EPO‐receptor binding leads to the phosphorylation of cytoplasmic domain‐associated JAK2 proteins (Constantinescu et al. 1999; Remy et al. 1999), which in turn provokes STAT5 phosphorylation and translocation to the nucleus where it activates gene transcription (Damen et al. 1993; Pallard et al. 1995; Penta and Sawyer 1995; Klingmuller et al. 1996; Quelle et al. 1996). In addition, EPO activation of JAK2 activates signaling cascades, such as the Ras/mitogen‐activated kinase (MAPK) pathway and the phosphatidylinositol 3‐kinase (PI3)/Akt pathway (Damen et al. 1993; Miura et al. 1994; Constantinescu et al. 1999; Fisher 2003). Several nonhematopoietic functions have been proposed for EPO, including protection against oxidative stress in neuronal cells (Zaman et al. 1999), neovascularisation in uterine angiogenesis (Yasuda et al. 1998) and myocardium maintenance and repair (Tada et al. 2006). Nonhematopoietic EPO functions are also mediated by the EPO‐R, which is consequently expressed in numerous nonhematopoietic tissues, including endothelial, neural, muscle, cardiovascular and renal tissues, and is activated in response to physical or metabolic stress (previously reviewed in Noguchi et al. 2008).

The pleiotropic roles of EPO have led to studies investigating its potential role in controlling skeletal muscle development and function. Ogilvie et al. (2000) first reported the presence of the *EPO*‐*R* mRNA and protein in proliferating mouse C2C12 myoblasts and primary satellite cells. More recently, several groups provided evidence of EPO‐R gene and/or protein expression in human primary myoblasts and satellite cells (Rundqvist et al. 2009; Launay et al. 2010) as well as in human muscle tissue (Lundby et al. 2008; Rundqvist et al. 2009; Christensen et al. 2012). However, it is unknown if the EPO‐R activates the same signaling cascades in skeletal muscle as in hematopoietic cells. While mouse C2C12 myoblasts treated with EPO displayed increases in JAK2, STAT5 (Ogilvie et al. 2000) and Akt phosphorylation (Jia et al. 2012), such effects could not be confirmed in rodent or human skeletal muscle in vivo (LeBaron et al. 2007; Christensen et al. 2012). In line with its role in erythroid progenitor cells, EPO promotes proliferation and survival and reduces differentiation in mouse myoblasts (Ogilvie et al. 2000; Jia et al. 2009). However, these effects could not be repeated in human or rat myoblasts (Rotter et al. 2008; Launay et al. 2010).

This lack of consistency across tissues and species highlights the need for further systematic investigations. Therefore, the aims of this study were to: first, thoroughly examine the expression levels of the EPO‐R gene and protein in mouse C2C12 and in human primary muscle cells during muscle cell proliferation and differentiation. Second, establish the effect of EPO treatment on human primary muscle cell proliferation, differentiation, as well as the ability of EPO to induce EPO‐R expression and to activate the JAK2/STAT5, the Akt and the MAPK signaling pathways in differentiated myotubes. Finally, as EPO may activate Akt signaling, a key pathway involved in muscle cell growth (Rommel et al. 2001), we investigated for the first time the effect of EPO treatment on protein synthesis in mouse C2C12 myotubes.

## Materials and Methods

### Cell culture and EPO treatment

Mouse C2C12 myoblasts (ATCC, Manassas, VA) were plated in six‐well tissue culture plates in complete Dulbecco's Modified Eagle's Medium (DMEM) supplemented with 10% fetal bovine serum (FBS) (Life Technologies, Mulgrave, Australia) and maintained in humidified air at 37°C and 5% CO_2_. As the cultures approached confluence, medium was changed to DMEM supplemented with 2% horse serum (HS) (Life Technologies). Differentiation medium was replaced every 48 h.

Human primary myoblasts were plated in six‐well tissue culture plates previously coated with an extracellular matrix (ECM) (Sigma, Castle Hill, Australia) in Hams F‐10 medium (Life Technologies) containing 20% FBS, 25 ng/mL fibroblast growth factor (bFGF) (Promega, Madison, WI), 0.05% penicillin/streptomycin (Life Technologies) and 0.05% amphoteromycin (Life Technologies). Cells were maintained in humidified air at 37°C and 5% CO_2_. At about 70% confluence, differentiation was induced by replacing the medium by DMEM supplemented with 2% HS. Differentiation medium was replaced every 48 h.

All cell culture experiments were performed at least in duplicate, using epoetin‐*α* (EPO Recombinant Human Protein, Life Technologies) and epoetin‐*β* molecules (NeoRecormon^®^, Roche Australia, Dee Why, Australia) at a concentration of 5 IU/mL, unless specified differently. Both epoetin‐*α* and ‐*β* have been previously used for in vitro EPO treatment at similar concentrations (Ogilvie et al. 2000; Rotter et al. 2008; Launay et al. 2010; Jia et al. 2012). For cell culture experiments, sample size was *n* = 3 unless specified differently. An effect of medium change on increasing the phosphorylation of selected proteins was investigated by replacing the differentiation medium with fresh medium containing a vehicle control. To avoid acute stress effects of medium replenishment, cells were serum starved for 12 h prior to EPO treatment with EPO then directly spiked into the medium. Acute EPO treatment experiments were paralleled by a negative control experiment where a vehicle control was spiked into the medium. Both EPO and vehicle control‐treated cells were harvested at the same time points.

### DNA sequencing

DNA sequencing was performed using the BigDye Terminator v3.1 Cycle Sequencing Kit (Applied Biosystems, Life Technolgies, Mulgrave, Australia) according to the manufacturer's instructions. DNA sequencing products were then separated using capillary electrophoresis (ABI 3130xl Genetic Analyser, Applied Biosystems, Life Technolgies).

### Agarose gel electrophoresis

Agarose gels containing 0.1 *μ*L/mL of SYBR^®^ Safe DNA Gel Stain (Life Technologies) were prepared by using 10 mg/mL DNA Grade Agarose (Life Technologies) in Tris‐acetate‐EDTA (TAE) buffer. DNA samples were mixed with the Gel Loading Dye Orange (New England Biolabs, Ipswich, MA) and loaded into the wells alongside a 1 kb Plus DNA Ladder (Life Technolgies). Electrophoresis was performed at 100 V and bands were visualized using a UV transilluminator (Vilber Lourmat, Marne la Vallée, France).

### RNA extraction and real‐time PCR

RNA was extracted using the Tri‐Reagent^®^ Solution (Ambion Inc., Austin, TX) according to the manufacturer's protocol. RNA concentration was assessed using the Nanodrop 1000 Spectrophotometer (Thermo Fisher Scientific, Waltham, MA). First‐strand cDNA was generated from 1 *μ*g RNA in 20 *μ*L reaction buffer using the High Capacity RT‐kit (Life Technolgies); 1× RT buffer and random primers, 8 mmol/L dNTP and 2.5 U/*μ*L MultiScribe™ RT enzyme. The RT protocol consisted of 10 min at 25°C, 120 min at 37°C, 5 min at 85°C then cooled to 4°C.

Real‐time PCR was carried out using a Stratagene MX3000 thermal cycler (Agilent Technologies, Santa Clara, CA). mRNA levels were measured using 1× SYBR^®^ Green PCR Master Mix (Agilent Technologies) and 5 ng of cDNA. All primers were used at a final concentration of 300 nmol/L. Primer sequences are presented in Table 1. The PCR condition conditions were 1 cycle of 10 min at 95°C; 40 cycles of 30 sec at 95°C, 60 sec at 60°C, 60 sec at 72°C; 1 cycle (melting curve) 60 sec at 90°C, 30 sec at 55°C, 30 sec at 95°C. To compensate for variations in input RNA amounts and efficiency of the reverse transcription, data were normalized to ribosomal protein *36B4* (also known as *RPLPO*) mRNA levels.

**Table 1.** List of primers used for RT‐PCR.

Gene (alias)	Human GenBank accession number Sequence 5′–3′	Mouse GenBank accession number Sequence 5′–3′
*EPOR (EPO‐R)*	NM_000121.3	NM_010149.3
	Sense GAG CAT GCC CAG GAT ACC TA	Sense CCC AAG TTT GAG AGC AAA GC
	Anti CAT GGC CAC TAT GTC CAC AC	Anti TGC AGG CTA CAT GAC TTT CG
*CKTM2*	NM_0010099735.1	NM_198415.2
	Sense ACG CAC TGG CCG AAG CAT CC	Sense ACG CAC TGG CCG AAG CAT CC
	Anti GCC AGA TCG CCC TTC AGG CC	Anti GCC AGA TCG CCC TTC AGG CC
*MYH1 (MHC1)*	NM_000257.2	NM_080728.2
	Sense ACC CTC AGG TGG CTC CGA GA	Sense ACC CTC AGG TGG CTC CGA GA
	Anti TGC AGC CCC AAA TGC AGC CA	Anti TGC AGC CCC AAA TGC AGC CA
*MYH2 (MHC2a)*	NM_001100112.1	NM_001039545.2
	Sense GAT GGC ACA GAA GTT GCT GA	Sense GAG CAA AGA TGC AGG GAA AG
	Anti CTT CTC GTA GAC GGC TTT GG	Anti TAA GGG TTG ACG GTG ACA CA
*MYH4 (MHC2x)*	NM_005963.3	NM_030679.1
	Sense AAG AGC AGG GAG GTT CAC AC	Sense GGA CCC ACG GTC GAA GTT GCA
	Anti TTA TCT CCA AAA GTC ATA AGT ACA	Anti GGA ACT CAT GGC TGC GGG CT
*VEGFA*	NM_001025366.2	NM_001025250.3
	Sense GCG CAA GAA ATC CCG GTA TA	Sense AAG CCA GCA CAT AGG AGA GAT GA
	Anti GCT TTC TCC GCT CTG AGC AA	Anti TCT TTC TTT GGT CTG CAT TCA CA
*MYOD*	NM_002478.4	NM_010866.2
	Sense CGT CGA GCA ATC CAA ACC A	Sense CTG CTT CTT CAC GCC CAA A
	Anti CTG CAG GCC CTC GAT ATA GC	Anti CTG GAA GAA CGG CTT CGA AAG
*MYC (c‐MYC)*	NM_002467.4	NM_001177352.1
	Sense TCG GGA AGT GGG AAA GCA	Sense CCC AAA TCC TGT ACC TCG TC
	Anti ATA GTT CCT GTT GGT GAA CCT AACG	Anti GCG TAG TTG TGC TGG TGA GT
*36B4*	NM_001002.3	NM_007475.5
	Sense TTG TGG GAG CAG ACA ATG TG	Sense TTG TGG GAG CAG ACA ATG TG
	Anti AGT CCT CCT TGG TGA ACA CG	Anti AGT CCT CCT TGG TGA ACA CG

### Protein extraction and western blot

Total protein was extracted using RIPA buffer (Millipore, North Ryde, Australia) with 1 *μ*L/mL protease inhibitor cocktail (Sigma, Castle Hill, Australia) and 10 *μ*L/mL Halt Phosphatase Inhibitor Single‐Use Cocktail (Thermo Scientific, Rockford, IL). Total protein content was determined using the BCA Protein Assay Kit (Pierce Biotechnology, Rockford, IL) according to the manufacturer's instructions.

Electrophoresis was performed using a 4–12% NuPAGE^®^ Novex Bis‐Tris Gel (Life Technolgies) in NuPAGE^®^ SDS MOPS Running Buffer (Life Technolgies). Protein transfer was performed in a Bjerrum buffer containing 50 mmol/L Tris, 17 mmol/L glycine and 10% methanol using PVDF membranes. The membranes were blocked with 5% BSA in PBS, after which they were incubated at 4°C with the following primary antibodies EPO‐R (M‐20, sc‐697, Santa Cruz Biotechnology, Santa Cruz, CA), phospho‐Akt (Ser473) (#9271, Cell Signalling Technology, Beverly, MA), phospho‐JAK2 (Tyr1007/Tyr1008) (sc‐21870, Santa Cruz Biotechnology, CA), phospho‐STAT5 (pY694) (Life Technolgies), phospho‐Erk1/2 (Thr202/Tyr204) (#4377, Cell Signalling Technology). The primary antibodies were diluted 1:1000 in PBS containing 5% BSA, except for phospho‐STAT5, which was diluted 1:500 in PBS containing 5% skim milk. Following overnight incubation, the membranes were washed and incubated for 1 h with an anti‐rabbit IgG antibody labeled with an infrared‐fluorescent 800 nm dye (Alexa Fluor^®^ 800, Life Technolgies) diluted 1:5000 in PBS containing 50% Odyssey^®^ blocking buffer (LI‐COR Biosciences, Lincoln, NE) and 0.01% SDS. Membranes exposed to the anti‐phospho‐JAK2 were incubated for 1 h with an anti‐goat IgG antibody labeled with an infrared‐fluorescent 488 nm dye (Alexa Fluor^®^ 488, Life Technolgies) diluted 1:5000 in PBS containing 50% Odyssey^®^ blocking buffer (LI‐COR Biosciences) and 0.01% SDS. After washing, the proteins were exposed on an Odyssey^®^ Infrared Imaging System (LI‐COR Biosciences) and individual protein band optical densities were determined using the Odyssey^®^ Infrared Imaging System software. All blots were normalized against the GAPDH protein (G8795, Sigma‐Aldrich, Sydney, Australia).

### Protein sequencing

#### Isoelectric fractionation of human myotube lysates

One milligram total protein from human myotube lysates was precipitated in acetone as previously described (Stephens et al. 2010) and resuspended in SB#2 buffer (urea 7 mol/L, thiourea 2 mol/L, C7BzO 1% w/v). Intact proteins were separated by isoelectric point using an Agilent 3100 OFFGEL fractionator (Agilent Technologies) according to the manufacturer's instructions. Focusing was carried out in 24 cm pH 3‐10 Immobiline™ DryStrip (GE Healthcare, Uppsala, Sweden) at a constant 50 *μ*A for a total of 64,000 volt hours. On completion, proteins from each fraction were collected and analyzed by western blot for the presence of EPO‐R. Fractions of interest were concentrated using a 2D Cleanup kit (BioRad, Hercules, CA) according to the manufacturer's instructions and resuspended in 80 *μ*L of 2× Laemeli SDS‐PAGE loading buffer. Samples were snap‐frozen and stored in 20 *μ*L aliquots at −80°C prior to analysis.

#### SDS‐PAGE and western blot analysis

Offgel‐concentrated fractions were separated by SDS‐PAGE using precast Criterion™ TGX Stain‐free™ 4–20% polyacrylamide gels (BioRad). To determine the location of bands reacting with EPO‐R antibody, partial transfer of proteins to low‐fluorescence PVDF membrane (Millipore, North Ryde, Australia) was undertaken for 90 sec at a constant 2.5 Å using a TransBlot^®^ Turbo™ system (BioRad). This limited transfer time was empirically determined to transfer sufficient protein to the membrane for western blotting, while leaving the remaining protein in the SDS‐PAGE gel for subsequent analysis by mass spectrometry. Blocking primary and secondary antibody conditions were the same as described above. Proteins remaining in the SDS‐PAGE gel were Coomassie‐stained overnight. To facilitate matching between images, a minimum of three separate lanes in each gel contained colorimetric protein standards (Precision Plus molecular weight markers; BioRad), which were easily detected in both gels and western blots following partial transfer.

Coomassie‐stained gels were scanned using a ProPicII robotic spotting platform (Genomic Solutions, Ann Arbor, MI), and both the gel and western blot images imported into Progenesis PG240 SameSpots software (Non Linear Dynamics, Newcastle, UK). Triangulation was performed using the molecular weight markers to accurately align both images. The location of bands detected by western blot was translated to the gel and exported directly to the ProPic II robot as a series of *x‐y* coordinates. Bands containing the proteins of interest were subsequently excised and deposited into microplates for digestion and mass spectrometric analysis.

#### LC‐MS/MS analysis of EPO‐R bands excised from SDS‐PAGE gels

All proteins underwent tryptic digestion overnight using standard procedures and MS/MS analysis and database searching were as previously described (Rainczuk et al. 2014). Spectra were subsequently interrogated for the presence of peptides corresponding to the full‐length or soluble forms of EPO‐R (UniPROT acc. P19235), using BioTools 3.2SR3 software (version 3.2 build 5.65; Bruker Daltonics, Bremen, Germany). The following search parameters were allowed; enzyme trypsin, maximum two missed cleavages; fixed modifications carbamidomethylation (C), variable modifications oxidation (M); 0.1 Da MS tolerance; 0.8 Da MS/MS tolerance.

#### Protein synthesis assay

Protein synthesis was determined by measuring the incorporation of radio‐labeled [^3^H]‐tyrosine (GE Healthcare, Sydney, Australia) into EPO‐treated or control mouse C2C12 myotubes (modified from Plaisance et al. 2008). Mouse C2C12 myotubes were also treated with 10 *μ*mol/L dexamethasone (DEX) (Sigma‐Aldrich, St. Louis, MO) to induce catabolic stress or 100 nmol/L insulin (INS) to induce anabolic stress. One *μ*Ci/mL of radio‐labeled [^3^H]‐tyrosine and 2 mmol/L l‐tyrosine (Sigma‐Aldrich) were added to the myotubes. The use of excess nonradioactive tyrosine in the medium gives an accurate indication of protein synthesis without alterations in the free intracellular tyrosine pool (Vandenburgh et al. 1989). Following this, myotubes were washed twice with cold phosphate‐buffered saline (PBS) and 1 mL of cold 10% trichloroacetic acid (TCA) (Sigma‐Aldrich) was added to each well. After scraping, myotubes sat on ice for 1 h to precipitate the proteins, followed by centrifugation at 20,000*g* for 10 min. The supernatant was removed and the precipitates were dissolved in 0.1 mol/L NaOH containing 1% Triton X‐100 (TX‐100) (Sigma‐Aldrich) overnight at room temperature. Following this, 400 *μ*L of the sample was mixed with 4 mL of Ultima Gold scintillation liquid (Perkin Elmer, Boston, MA) and TCA soluble radioactivity was measured using a Wallac1409 DSA liquid scintillation counter (Perkin Elmer), as previously described by our group (Foletta et al. 2013).

#### Proliferation assay

The rate of mouse C2C12 myoblast proliferation with and without EPO treatment was assessed using the colorimetric 5‐bromo‐2′‐deoxy‐uridine (BrdU) Labeling and Detection Kit III assay (Roche Applied Science, Indianapolis, IN) according to the manufacturer's protocol. Briefly, cells were plated at a concentration of 10^4^ cells/mL in a 96‐well tissue culture plate. The BrdU label reagent was added to the medium for 24 h prior to each time point analysis. Nucleases were then used to fragment DNA and BrdU incorporation into the DNA was detected using a horseradish peroxidase conjugated anti‐BrdU antibody. Following the addition of the peroxidase substrate ABTS (2,2′‐azino‐bis, 3‐ethylbenzthiazoline‐6‐sulfonic acid), fluorescence was detected at 405 nm.

### Statistical analysis

All data are reported as mean ± SEM. Gene and protein expression during differentiation, myoblast proliferation and protein synthesis were analyzed using a one‐way analysis of variance (ANOVA) or a two‐way ANOVA. Significant pairwise differences were detected using the Student–Newman–Keul's post hoc test. All other data were analyzed using a two‐tailed unpaired *t*‐test. The level of significance was set at *P* < 0.05.

## Results

### *EPO*‐*R* gene expression in muscle cells

**Figure 1.**
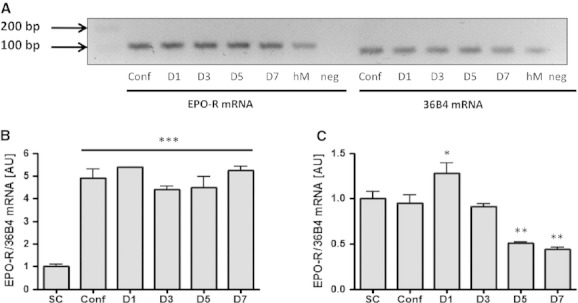
*EPO*‐*R* gene expression during differentiation of mouse C2C12 cells or human primary muscle cells measured by (A) agarose gel electrophoresis and (B, C) RT‐PCR. (A) Agarose gel representing human *EPO*‐*R* transcript expression in primary human skeletal muscle cells during differentiation and in human muscle tissue extracts. Conf, confluent myoblasts; D1, day 1 after differentiation into myotubes; hM, human muscle tissue extract; Neg, no cDNA control. *EPO*‐*R* gene expression during differentiation: (B) mouse C2C12 cells, (C) human primary muscle cells. SC, subconfluent myoblasts; Conf, confluent myoblasts; D1, day 1 after differentiation into myotubes. Data are represented as mean ± SEM. Significantly different from SC, *P < 0.05, **P < 0.01, ***P < 0.001.

DNA sequencing returned sequences corresponding to the mouse and human *EPO*‐*R* transcripts, respectively (data not shown). The *EPO*‐*R* transcript was detected at each stage of proliferation and differentiation in both mouse C2C12 and human primary muscle cells. A transcript of the same size was also detected in lysates from human skeletal muscle tissue (Fig. 1A). *EPO*‐*R* mRNA levels were increased by approximately fivefold in confluent mouse C2C12 myoblasts when compared to subconfluent myoblasts. This level of expression was then sustained during myoblast to myotube differentiation (Fig. 1B). Inversely, *EPO*‐*R* mRNA levels decreased by about twofold as human primary myoblasts terminally differentiated into myotubes (Fig. 1C).

### Identification and expression of the EPO‐R protein in muscle cells

**Figure 2.**
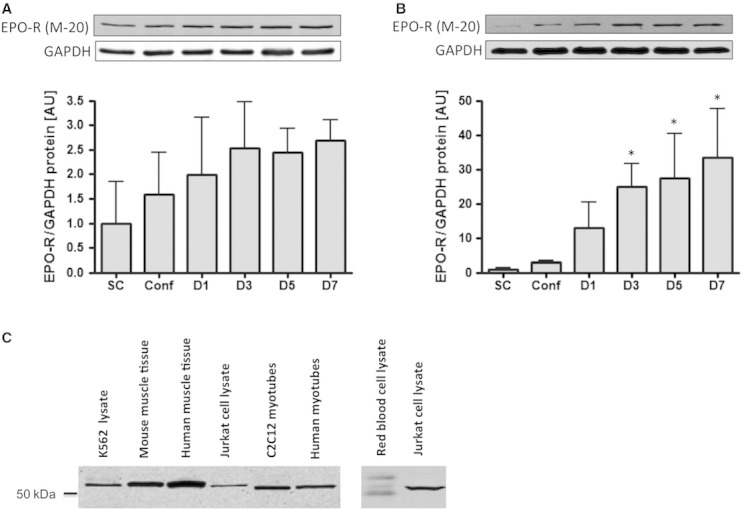
EPO‐R protein expression during differentiation of mouse C2C12 cells or human primary muscle cells. (A) Mouse C2C12 cells and (B) human primary muscle cells. SC, subconfluent myoblasts; Conf, confluent myoblasts; D1, day 1 after differentiation into myotubes. Data are represented as mean ± SEM. *Significantly different from SC,* P* < 0.05. (C) EPO‐R protein detection by western blotting using the M‐20 antibody (sc‐697, Santa Cruz Biotechnology).

**Figure 3.**
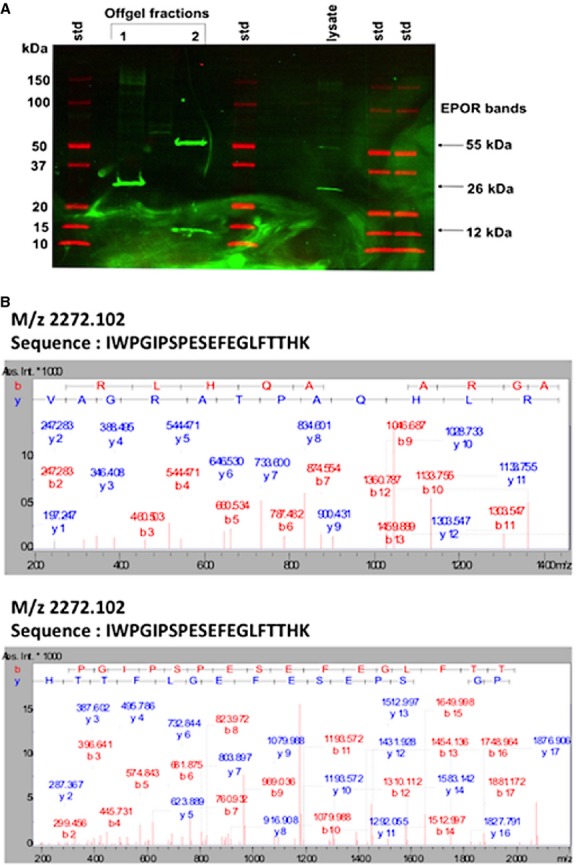
Analysis of bands detected by the M‐20 EPO‐R antibody (sc‐697, Santa Cruz Biotechnology). (A) Protein bands from human myotube lysates that reacted with the anti‐EPO‐R antibody were identified by western blotting. Three consistently observed bands at ~55, ~26 and ~12 kDa were analyzed by mass spectrometry. Multiple lanes of standards were used for image triangulation during spot cutting. (B) Example MS/MS spectra for peptides identified from the ~55 kDa full‐length EPO‐R (upper panel) or the ~26 kDa soluble EPO‐R (lower panel, unique to the soluble form). Additional peptides are listed in Appendix 1.

Following western blotting using the EPO‐R antibody (M‐20, sc‐697, Santa Cruz Biotechnology), a single band located slightly above 50 kDa corresponding to the full‐length EPO‐R protein was detected at each stage of mouse C2C12 and human primary muscle cell development. The level of expression of the EPO‐R protein increased during differentiation in both types of cells, although this increase was more dramatic in human primary muscle cells than in mouse C2C12 muscle cells. The amount of EPO‐R protein in fully differentiated human myotubes was about 30‐fold greater than in myoblasts, whereas this increase was about threefold in mouse C2C12 myotubes (Fig. 2A and B). Additionally, the antibody also detected a 50 kDa band in K‐562 cells extracts, Jurkat cell lysates and in red blood cell extracts, which are all commonly used as positive controls for EPO‐R expression (Fig. 2C). To establish the specificity of the antibody to detect the EPO‐R protein, fractionation of myotube lysates by isoelectric point and concentration of the relevant fractions revealed three distinct bands (~55, 26, and 12 kDa) that reacted with the EPO‐R antibody (Fig. 3A). Each of these bands was excised from gels and analyzed by LC‐MS/MS for the presence of peptides corresponding to the EPO‐R. In each case, multiple tryptic peptides matching the EPO‐R were observed (Fig. 3B and Appendix 1). Moreover, further analysis revealed that in the case of the 26 kDa reactive band, two of the identified peptides corresponded to the C‐terminal region (amino acids 196–209) of the soluble form of the EPO‐R (Fig. 3B lower panel and Appendix 1). The data suggest that both the membrane‐bound and soluble forms of the EPO‐R are present in human myotube lysates.

### The effect of EPO treatment on *EPO*‐*R* gene expression in muscle cells

**Figure 4.**
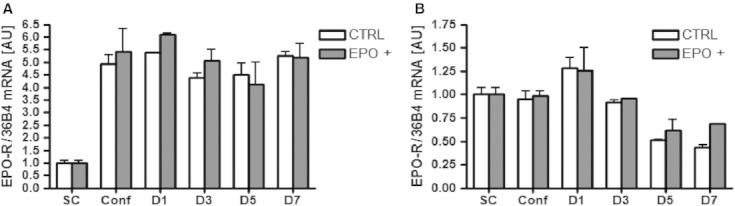
*EPO*‐*R* expression measured by RT‐PCR during differentiation of mouse C2C12 cells or human primary muscle cells treated with EPO. EPO treatment does not influence *EPO*‐*R* gene expression during muscle cell differentiation. (A) Mouse C2C12 cells and (B) human primary muscle cells. Data are represented as mean ± SEM. Statistical analysis revealed no differences between groups at any of the time points.

Five IU/mL EPO treatment had no effect on the *EPO*‐*R* gene expression during mouse C2C12 or human primary muscle cell proliferation or differentiation (Fig. 4). The same result was observed for EPO‐R protein expression (data not shown).

### The effect of EPO treatment on mouse C2C12 myoblast proliferation

**Figure 5.**
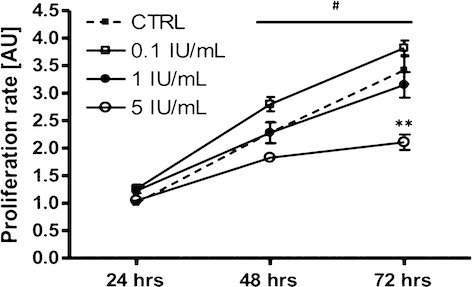
BrDU proliferation assay. On mouse C2C12 myoblast proliferation, 24 h, 48 h and 72 h EPO treatment has no effect. Sample size was *n* = 6 for each group. Experiment was performed in duplicate. Data are represented as mean ± SEM. #Significantly different from 24 h, *P* < 0.001. **Significantly different from CTRL,* P* < 0.01.

Treatment with EPO for 24, 48 and 72 h at 0.1 and 1 IU/mL had no effect on mouse C2C12 myoblast proliferation when compared to untreated cells. However, 5 IU/mL significantly attenuated myoblast proliferation after 72 h of treatment when compared to control cells (*P* < 0.01; Fig. 5).

### The effect of EPO treatment on muscle cell differentiation

Five IU/mL EPO treatment had no effect on the mRNA levels of the muscle differentiation markers *CKMT2, MHCI*,* MHCIIa*, and *MHCIIx*, the angiogenic factor marker *VEGFA*, the myogenic factor *MYOD* and the cell cycle regulator *c‐MYC* at any stage of mouse C2C12 or human primary muscle cell proliferation and differentiation (data not shown).

### The effect of medium replenishment on molecular signaling

**Figure 6.**
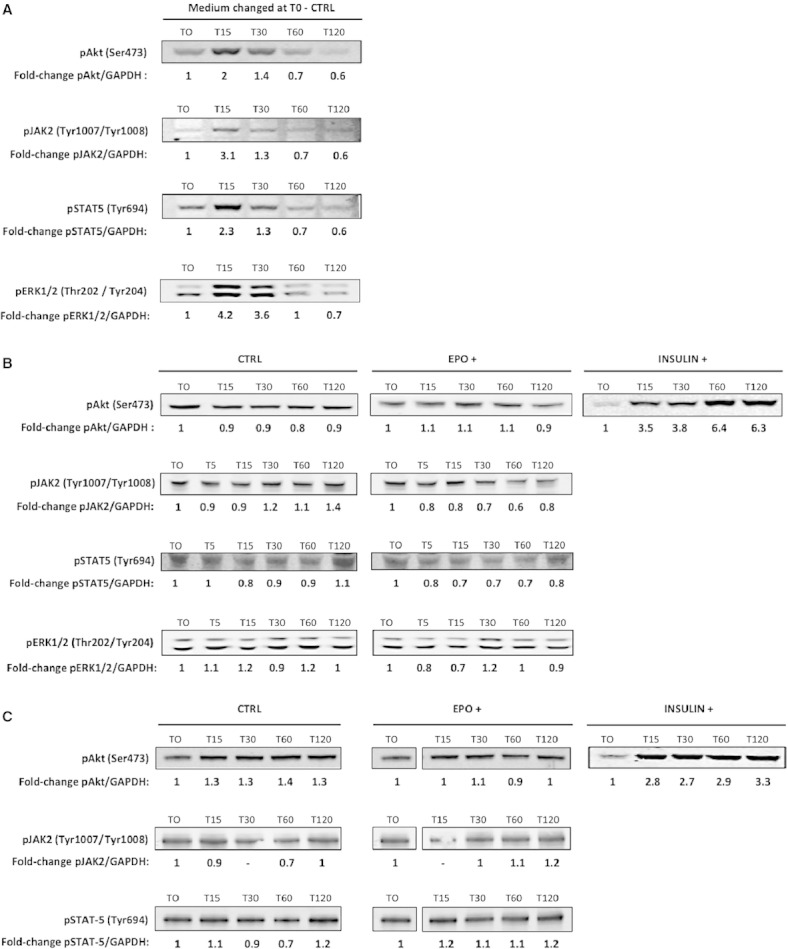
Medium replenishment but not acute EPO treatment phosphorylates Akt, JAK2, STAT5 and pERK1/2 in muscle cells. Fold‐change refers to the AU values normalized against GAPDH. (A) Effect of medium replenishment in mouse C2C12 cells, (B) effect of acute EPO treatment in mouse C2C12 cells, (C) effect of acute EPO treatment in human primary muscle cells.

Replenishment of fresh medium can introduce cell stress that may activate growth regulating kinases. This stress may be mechanical, from removing and replenishing the medium, or nutrient, from the fresh medium containing cytokines and growth factors. Following medium change in mouse C2C12 myotubes, Akt, JAK2, STAT5 and ERK1/2 displayed an increase in phosphorylation at 15 and 30 min and had returned back to basal levels after 60 min (Fig. 6A). This effect was also observed, albeit to a smaller extent, in human primary myotubes (data not shown).

### The effect of EPO treatment on molecular signaling

To avoid stress caused by changing media, cells were serum starved for 12 h before spiking EPO or a vehicle directly into the medium. Fifteen, 30, 60 and 120 min of EPO spike‐in treatment had no effect on Akt phosphorylation in mouse C2C12 myotubes when compared to cells treated with a vehicle control. Five, 15, 30, 60 and 120 min of EPO spike‐in treatment had no effect on JAK2, STAT5 and ERK1/2 phosphorylation either (Fig. 6B). Insulin treatment phosphorylates Akt and this was used as a positive control to demonstrate that the myotubes were responsive. A 3.5‐ to 6.3‐fold increase in Akt phosphorylation was observed between 15 and 120 min of insulin treatment in mouse C2C12 myotubes.

Similarly, in human primary myotubes, 15, 30, 60 and 120 min of EPO spike‐in treatment had no effect on Akt, JAK2, or STAT5 phosphorylation when compared to cells treated with a vehicle control (Fig. 6C). Human primary myotubes were sensitive to insulin as indicated by an increase in Akt phosphorylation.

The same phenomenon was observed in mouse C2C12 and in human primary myoblasts where EPO spike‐in treatment had no effect on Akt activation (data not shown).

### The effect of EPO treatment on protein synthesis in mouse C2C12 myotubes

**Figure 7.**
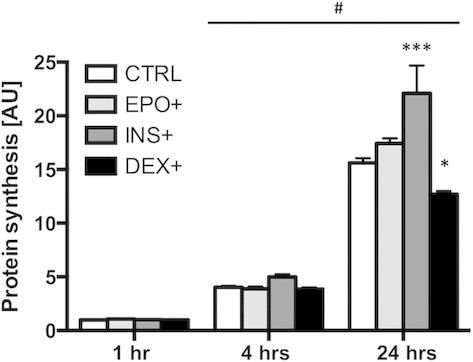
Protein synthesis assay. EPO treatment has no effect on protein synthesis in mouse C2C12 myotubes. Sample size was *n* = 6 for each group. Experiment was performed in duplicate. Data are represented as mean ± SEM. #Significantly different from 1 h, *P* < 0.001. ***Significantly different from CTRL,* P* < 0.001. *Significantly different from CTRL,* P* < 0.05.

One, 4 and 24 h of treatment with 5 IU/mL EPO had no effect on protein synthesis in mouse C2C12 myotubes when compared to untreated cells (Fig. 7). Insulin (INS) and dexamethasone (DEX) increase and decrease muscle protein synthesis, respectively, and were used as controls to demonstrate that the cells were responsive to anabolic and catabolic stimuli. As expected, 24 h of insulin treatment induced anabolic stress and significantly augmented protein synthesis by 1.4‐fold (*P* < 0.001), whereas 24 h of treatment with the catabolic agent dexamethasone significantly decreased protein synthesis by 1.25‐fold (*P* < 0.05).

## Discussion

Erythropoietin has been considered as positive regulator of skeletal muscle myogenesis and repair (Noguchi et al. 2008), however, numerous conflicting observations exist within the field. Contributing to inconsistencies between studies have been the use of muscle cells from different species, comparisons made between in vitro and in vivo experimental models and the use of nonspecific antibodies (see Lamon and Russell 2013). This study aimed to clarify some of these inconstancies and several new observations were made. First, *EPO*‐*R* gene expression was confirmed in both mouse C2C12 and human primary skeletal muscle myoblasts and myotubes using real‐time PCR followed by DNA sequencing. Second, for the first time, western blotting followed by the use of LC‐MS/MS identified that the M‐20 clone EPO‐R antibody (Santa Cruz) recognizes the EPO‐R in mouse C2C12 and human myoblast and myotube extracts. Third, spike‐in treatment of mouse C2C12 and human primary muscle cells with EPO did not regulate proliferation, differentiation or protein synthesis. Finally, we observed that Akt, JAK2, STAT5 and ERK1/2 phosphorylation is sensitive to the stress of changing culture medium and not due to EPO treatment.

In mouse C2C12 cells, *EPO*‐*R* mRNA levels were upregulated during myoblast proliferation, a result in accordance with those of Ogilvie et al. (2000). However, in this study the upregulation of *EPO‐R* mRNA levels was maintained during myoblast differentiation, in contrast to results previously reported by Jia et al. (2012). Interestingly, we observed the opposite pattern in human primary skeletal muscle cells. Here *EPO*‐*R* mRNA levels were highly expressed during myoblast proliferation but gradually decreased during differentiation into mature myotubes. This pattern of *EPO*‐*R* mRNA regulation in human primary skeletal muscle cells is analogous to that observed during erythropoiesis, where the EPO‐R is not expressed in mature red blood cells (Krantz 1991).

Expression of the EPO‐R protein in skeletal muscle is a contentious topic, mainly due to a wide controversy surrounding the nonspecificity of certain commercially available EPO‐R antibodies (previously reviewed in Lamon and Russell 2013). A study comparing several commercially available EPO‐R antibodies suggested that the M‐20 clone (sc‐697, Santa Cruz Biotechnology) was the only antibody suitable for detection of the EPO‐R protein by western blot known to date (Elliott et al. 2006), although the same group recently reported that, in a breast tumor cell line (MCF‐7), the M‐20 antibody also bound to a nonspecific protein migrating at a similar molecular weight as the EPO‐R (Elliott et al. 2013). Accordingly, we report that the M‐20 clone recognizes a single band at the predicted size of the EPO‐R in human skeletal muscle and mouse C2C12 cell extracts. In order to assess the specificity of this band with the greatest possible certainty, we performed mass spectrometry on those proteins reacting with the EPO‐R antibody. LC‐MS/MS analysis revealed peptides corresponding to the sequences of both the full‐length and soluble forms of human EPO‐R. Our results confirm for the first time the presence of the EPO‐R protein at each stage of myogenesis in mouse C2C12 and human skeletal muscle cells. In both mouse and human muscle cells, the protein detected by the M‐20 antibody was upregulated during differentiation. While this regulatory pattern was similar to *EPO*‐*R* gene expression in mouse C2C12 cells during differentiation, the opposite was observed in human primary skeletal muscle cells. The discrepancies existing between gene and protein expression in human primary skeletal muscle cells, and also between mouse C2C12 and human muscle cells, may reflect a different function of EPO and its receptor in the two species. This is not without precedent in the literature. In vivo, high‐EPO dose injections increased mouse exercise performance independently of erythropoiesis but via the presence of EPO in the brain (Schuler et al. 2012); a result in contrast with human data (Rasmussen et al. 2010). Previous studies have shown that mouse C2C12 and human primary muscle cells can respond differently to external stimuli (Czifra et al. 2006; Dogra et al. 2006, 2007a,b; Owens et al. 2013; Sharma et al. 2013). Alternatively, the stability of the mature receptor protein may vary between mouse and human muscle cells. Finally, it cannot be excluded that the band recognized by the EPO‐R antibody contains more than one protein, as previously suggested with MCF‐7 cell lysates (Elliott et al. 2013), although the negative control used in this particular study has not been validated. However, in order to verify this hypothesis, another validated, commercially available EPO‐R antibody is required for the immunoprecipitation of the proteins. In addition, the lack of an easily accessible, validated negative control for the EPO‐R suggests that the protein expression results measured via western blot should be considered carefully, as a partial nonspecificity of the band may potentially account for the observed differences.

EPO‐R gene and protein expression was not altered by EPO stimulation at any stage of differentiation in mouse C2C12 and human primary skeletal muscle cells. This is in contrast to previous studies suggesting that EPO treatment triggers the expression of the EPO‐R gene and protein in mouse C2C12 myoblasts (Ogilvie et al. 2000; Jia et al. 2009), but supports observations that EPO administration does not regulate *EPO*‐*R* mRNA levels in human skeletal muscle (Lundby et al. 2008). In line with its role in erythroid progenitor cells, EPO is proposed to enhance proliferation and survival as well as reduce differentiation and fusion in mouse C2C12 cells (Ogilvie et al. 2000; Jia et al. 2009, 2012). However, we failed to observe an increase in mouse C2C12 myoblast proliferation following EPO treatment in our experimental conditions. Likewise, EPO treatment in mouse C2C12 or human primary skeletal muscle cells did not alter the expression of genes associated with skeletal muscle cell differentiation, including the myogenic factor* MyoD*. These results support those of Launay et al. (2010), who reported that EPO supplementation did not promote proliferation or postpone differentiation in rat or human myoblasts cultured in either normoxic or hypoxic conditions.

An increase in Akt signaling has been observed in cultured mouse C2C12 myoblasts following EPO treatment (Jia et al. 2012). Additionally, supraphysiological levels of circulating EPO activated Akt in mouse skeletal muscle (Hojman et al. 2009). Whether this activation was EPO‐R‐dependent has not been established. We consequently hypothesized that EPO may activate Akt in human skeletal muscle cells. Additionally, as Akt increases skeletal muscle hypertrophy, we were also interested to determine whether EPO can increase muscle cell protein synthesis (Rommel et al. 2001). We first observed that in mouse C2C12 and human myotubes, Akt, JAK2, STAT5 and ERK1/2 phosphorylation increased following medium refreshment; an increase that was independent of the presence of EPO in the medium. This effect of medium change on the phosphorylation of signaling molecules in muscle cells is not without precedent (Sinclair et al. 2010) and caution should therefore be taken when manipulating cell culture treatments, while methodology should be precisely stated. To eliminate the stress‐related increase in Akt, JAK2, STAT5 and ERK1/2 phosphorylation due to medium change, EPO or a vehicle control was spiked directly into the medium. EPO treatment did not lead to any change in Akt, JAK2 and STAT5 phosphorylation. EPO has the potential to trigger the MAPK pathway in interleukin‐3‐dependent cell lines expressing high levels of EPO‐R (Miura et al. 1994). However, we observed no alteration in ERK1/2 phosphorylation following EPO treatment in mouse C2C12 myotubes. Overall, these in vitro results support those recently reported showing that acute EPO administration did not lead to any change in the phosphorylation levels of members of the Akt and MAPK signaling pathways in human skeletal muscle in vivo (Christensen et al. 2012). EPO treatment did not lead to any change in protein synthesis in mouse C2C12 myotubes, although these cells responded to both anabolic (insulin) and catabolic stimuli (dexamethasone), a logical result considering there was no increase in Akt phosphorylation.

## Conclusion

In conclusion, we demonstrate here that EPO‐R mRNA and protein is expressed in both mouse C2C12 and human primary skeletal muscle myoblasts and myotubes. Our observations that EPO treatment, via a spike into the medium, failed to regulate muscle cell proliferation, differentiation or protein synthesis and did not influence EPO‐R expression or the phosphorylation levels of Akt, JAK2, STAT5 or ERK1/2 questions the direct role of EPO in muscle function; a question that has been previously raised by several research groups (Launay et al. 2010; Christensen et al. 2012). The use of overexpression and knockdown strategies targeting the EPO‐R in muscle cells or rodent models is therefore crucial to improve our understanding of the potential role that EPO and EPO‐R may play in skeletal muscle health.

## Conflict of Interest

None declared.

## Appendix

### A. Peptides identified by LC‐MS/MS corresponding to EPOR

Peak lists generated following LC‐MS were examined for the presence of peptides with expected masses corresponding to EPO‐R. Search paramaters: charge +1, MS tolerance 0.1 Da, MS/MS tolerance 0.8 Da, enzyme Trypsin, global modifications carbamidomethyl (C), optional modifications oxidation (M) (see methods section for a detailed description). The sequence of each EPO‐R isoform is provided, with the location of matched peptides shown in red.

#### B. Annotated MS/MS spectra

Annotated MS/MS spectra corresponding to matched peptide sequences are provided with b and y ion series shown in red or blue, respectively.
